# A Novel Experimental Approach to Understand the Transport of Nanodrugs

**DOI:** 10.3390/ma16155485

**Published:** 2023-08-05

**Authors:** Soubantika Palchoudhury, Parnab Das, Amirehsan Ghasemi, Syed Mohammed Tareq, Sohini Sengupta, Jinchen Han, Sarah Maglosky, Fajer Almanea, Madison Jones, Collin Cox, Venkateswar Rao

**Affiliations:** 1 Chemical and Materials Engineering, University of Dayton, Dayton, OH 45469, USA; 2 Civil, Construction and Environmental Engineering, The University of Alabama, Tuscaloosa, AL 35487, USA; 3 The Bredesen Center for Interdisciplinary Research and Graduate Education, University of Tennessee, 444 Greve Hall, 821 Volunteer Blvd., Knoxville, TN 37996-3394, USA; 4 Civil and Chemical Engineering, University of Tennessee, Chattanooga, TN 37403, USA

**Keywords:** metal oxide nanodrugs, iron oxide, zinc oxide, Cu-Zn-Fe oxide, transport of nanodrugs, material characterization

## Abstract

Nanoparticle-based drugs offer attractive advantages like targeted delivery to the diseased site and size and shape-controlled properties. Therefore, understanding the particulate flow of the nanodrugs is important for effective delivery, accurate prediction of required dosage, and developing efficient drug delivery platforms for nanodrugs. In this study, the transport of nanodrugs including flow velocity and deposition is investigated using three model metal oxide nanodrugs of different sizes including iron oxide, zinc oxide, and combined Cu-Zn-Fe oxide synthesized via a modified polyol approach. The hydrodynamic size, size, morphology, chemical composition, crystal phase, and surface functional groups of the water-soluble nanodrugs were characterized via dynamic light scattering, transmission electron microscopy, scanning electron microscopy-energy dispersive X-ray, X-ray diffraction, and fourier transform infrared spectroscopy, respectively. Two different biomimetic flow channels with customized surfaces are developed via 3D printing to experimentally monitor the velocity and deposition of the different nanodrugs. A diffusion dominated mechanism of flow is seen in size ranges 92 nm to 110 nm of the nanodrugs, from the experimental velocity and mass loss profiles. The flow velocity analysis also shows that the transport of nanodrugs is controlled by sedimentation processes in the larger size ranges of 110–302 nm. However, the combined overview from experimental mass loss and velocity trends indicates presence of both diffusive and sedimentation forces in the 110–302 nm size ranges. It is also discovered that the nanodrugs with higher positive surface charges are transported faster through the two test channels, which also leads to lower deposition of these nanodrugs on the walls of the flow channels. The results from this study will be valuable in realizing reliable and cost-effective in vitro experimental approaches that can support in vivo methods to predict the flow of new nanodrugs.

## 1. Introduction

Nanoparticles are highly promising for next-generation medical solutions ranging from new oncology drugs, contrast agents for magnetic resonance imaging (MRI), and vehicles for drug delivery [1,2,3,4]. A large section of nanoparticle-based drugs is used in cancer research with more than a dozen nanodrugs being approved for clinical use by the Federal Drug Administration (FDA) [5,6] For example, Doxil or Caelyx, a dextran-based iron oxide nanoparticle formulation was one of the first FDA-approved nanodrug for cancer treatment [7]. Feridex is another iron oxide nanoparticle-based formulation that has been approved for application as a contrast agent to enhance MRI images [8,9,10]. Feraheme has been FDA-approved for treatment of iron deficiency diseases [11,12]. Iron oxide-based nanoparticles are also highly attractive for hyperthermia treatment of cancer [13]. Zinc oxide nanoparticles are FDA-approved food additives and are known for their antimicrobial properties. One of the key advantages of nanoparticles is that a vast range of tunable properties can be achieved through the controlled size, shape, surface functionalization, and stiffness of the nanoparticles. The surfaces of the nanoparticles can be modified to bind with specific receptors or proteins expressed at the diseased site for targeted delivery of a drug. In other instances, the nanoparticle can also be carried to the tumor site via passive targeting using the enhanced permeability and retention (EPR) effect [14]. These examples highlight the tremendous potential of metal oxide-based nanodrugs in transformative medical solutions as well as drug delivery applications. Therefore, this study will primarily focus on metal oxide nanodrugs and will seek to understand their properties such as velocity and deposition on the walls during their transport through the body. Soft materials such as various polymeric and protein-based nanodrugs that have been reported in literature will be investigated as a separate class of materials in future studies [15,16,17].

Several challenges including complex pharmacokinetics, lack of reliable in vitro models, and time and cost required for in vivo rodent models impede preclinical to clinical translation rate of the new nanodrugs [18,19]. Rodents share 84% similarity with humans from a genomic perspective. Other in vivo models such as zebrafish share 76% of human genes while chickens share 80%. The drug discovery and screening process comprise multiple iterative in vivo trials on rodents or other small animals to understand the pharmacokinetics and toxicity of the new nanodrug that require time and precision. In addition, the existing success rate of clinical trials of nanodrugs is low. A 14% success rate is seen for phase 3 clinical trials of nanodrugs related to cancer treatment [20]. A lack of overall understanding of the interaction and transport of nanodrugs during circulation within the human body is one of the major causes of these failed clinical translations [18,21]. Further, the animal models may not be able to fully replicate all aspects of human disease. Therefore, an experimental model that can predict the transport of nanodrugs can serve as a key and cost-effective support for the in vitro and in vivo studies at the preclinical stages [22]. Such an experimental model can further bridge the gap between preclinical and clinical stages and can enhance the success rate from the bench to clinic for new nanodrugs. Several in vivo models such as endocytosis pathways for internalization of nanodrugs, nanotoxicity evaluation, evaluation of targeting specificity of the nanodrugs have been reported in literature, which can serve as a next phase of assessment, following the preliminary screening via the new experimental approach [23,24,25,26,27,28,29].

Traditionally, 2D cell culture models have been widely used for in vitro testing of various drug molecules. However, several 3D models including multicellular cancer spheroids, hydrogel scaffolds, and bioprinted engineered tissues have emerged as more effective alternatives for understanding the complex molecular transport and cellular interactions of nanodrugs [30]. For example, the multicellular spheroid models have been used to investigate size-dependent penetration of gold nanoparticles in human breast cancer as well as to image the penetration of quantum dots, albumin-based nanodrugs, iron oxide-based nanodrugs, and micelles in cervical cancer [31,32]. Hydrogel networks have been used in investigating the transport of silica nanoparticles as well as comparison of rigid and soft nanoparticles [15,16]. Engineered colon cancer spheroids have helped in studying the effect of surface charge of the nanodrugs on their penetration depth [28]. However, these models are specific to the cell type and require advanced imaging techniques. A more facile and cost-effective approach will serve as a major support to the advanced in vitro and in vivo studies for understanding the transport of new nanodrugs. 

To this end, different computational approaches have been reported for nanodrugs in an effort to translate the preclinical stage efficacy to clinical trials [33,34,35,36,37]. For example, Moreno-Chaparro et al., reported that the nanoparticle’s shape plays a key role in the transport behavior for surface functionalized nanoparticles [38]. The group used the translational and rotational diameter to determine this overall effect of nanoparticle shape on transport properties and a rigid multi-blob method to discretize the nanoparticle systems for solution. We have previously reported a combined computational and experimental approach to predict the velocity and deposition of nanodrugs through various types of flow channels including straight, circular, and hydrogel-based [39,40]. However, these flow channels did not account for the blood vessels or biomolecules that lie in the path of the nanodrugs in real vascular or pulmonary arterial environment [41]. 

Herein, a new experimental approach is reported to investigate the transport of three different new metal oxide nanodrugs. New geometries of the flow channels are designed with structures within the path of flow to replicate a biomimetic environment. These channels serve as reference flow paths for understanding the transport of nanodrugs. The effect of the size of the nanodrug on average velocity and deposition of the nanodrug during flow is investigated. The experimental approach is conducted with three new formulations of metal oxide nanodrugs reported here for the first time. The primary mechanisms dominating the transport of nanodrugs in the specific flow regime being investigated are realized from the experimental velocity and mass loss of the nanodrugs. 

## 2. Experimental Methods

### 2.1. Chemicals Used 

The chemicals were purchased from Sigma-Aldrich, Thermo Scientific, and Fisher, and used without further modification. These include deionized (DI, VWR) water, zinc (II) acetylacetonate (Zn(acac)_2_, Thermo Scientific), iron (III) 2,4-pentanedionate (Fe(acac)_3_, Alfa Aesar), copper (II) acetylacetonate (Cu(acac)_2_, ACROS, 98%), triethylene glycol (TREG, Thermo Scientific, 99%), poly(ethyleneimine) solution (PEI, Sigma-Aldrich, Mw: 60,000 kDa-50 wt% in water), and polyvinylpyrrolidone (PVP, Sigma Aldrich, Mw: 10,000 kDa). 

### 2.2. Synthesis of Nanodrugs

Three different nanodrug compositions including iron oxide, zinc oxide, and Cu-Zn-Fe oxide coated with biocompatible ligands were synthesized via a modified polyol method using a Schlenk line technique, similar to our previously reported protocol [39,40]. These three new metal oxide compositions served as model nanodrugs of varying sizes for subsequent experiments to investigate the transport of nanodrugs (Supplementary Materials, Figure S1). In a typical synthesis of iron oxide nanodrugs, a ligand mixture of PVP/PEI (0.06 mmol PVP/0.005 mmol PEI) was purged in nitrogen for one hour and dissolved in the solvent, TREG (10 mL) by heating at 90 °C for 10 min. The iron precursor, Fe(acac)_3_ (2 mmol) was then added to the reaction mixture and stirred for 30 min, prior to heating up at 290 °C for an hour to form the iron oxide nanodrugs. Unreacted chemicals and remnant organics were removed from the nanodrug product by precipitating the product via centrifugation (room temperature, 15,000 rpm, 15 min, Sorval^TM^ Legend^TM^ Micro 17 microcentrifuge, Fisher) using a washing mixture of ethanol and DI water. The sample was washed two times and the nanodrug precipitate was re-dissolved in DI water to form the final product. 

In a similar approach, the zinc oxide nanodrugs were synthesized by heating the PVP/PEI (0.06 mmol PVP/0.008 mmol PEI) ligand mixture (90 °C for 10 min) to dissolution in the solvent and reducing agent, TREG (10 mL). The Zn(acac)_2_ (2 mmol) was then added, and the reaction mixture was heated at 290 °C for one hour to form the zinc oxide nanodrugs. The sample was cleaned via centrifugation using ethanol/water mixture, following a procedure similar to that described for the iron oxide nanodrugs to obtain the final product.

The combined nanodrugs consisting of a combined oxide of copper, zinc, and iron was prepared via a similar procedure using 0.06 mmol of PEI as the ligand and 10 mL of TREG as the solvent and reducing agent. Precursor salts for the cations including Cu(acac)_2_ (0.25 mmol), Zn(acac)_2_ (0.25 mmol), and Fe(acac)_3_ (2 mmol) were added to the reaction mixture after complete dissolution of the ligand in the solvent. The reaction was heated at 290 °C for one hour to form the combined nanodrugs. The final nanodrug product was obtained by removing the remnant organics and unreacted chemicals via ethanol/DI water washes using centrifugation as described earlier. The cleaned nanodrug products were subsequently used for characterization of the size and surface properties. 

### 2.3. Characterization

The size and surface charge of the synthesized nanodrugs were investigated via a Brookhaven NanoBrook 90Plus particle analyzer dynamic light scattering (DLS) unit equipped with zeta potential capability. Aliquots of the three different nanodrug samples diluted in DI water to concentrations of 1.5 gL^−1^ were added in the polystyrene cuvettes for the DLS size analyses. Measurements were reported as an average of five consecutive runs at room temperature. Sizes of the nanodrugs were reported based on intensity-weighted DLS measurements. Samples for zeta potential measurements were prepared by adding 0.6 mL aliquots of the nanodrugs diluted in DI water (1.5 gL^−1^) to the SZP surface zeta potential cuvettes. The zeta potential of the nanodrug samples were recorded based on electrophoretic light scattering measurements at room temperature as an average of five consecutive runs. 

In addition, the size and morphology of the three different nanodrug samples were also investigated on a Hitachi H-7600 transmission electron microscope (TEM). Aqueous dispersions of the nanodrugs were mixed well via sonication (Branson 1800, Fisher) at room temperature for 15 min. Aliquots of these well mixed samples were dropped on 300 mesh copper TEM grids and air dried to prepare the samples for TEM imaging. 

A Phenom ProX Desktop Generation 5 scanning electron microscope (SEM) equipped with energy dispersive X-ray (EDX) was used to investigate the surface morphology and chemical composition of the three new nanodrug compositions. Dried samples of the nanodrugs for SEM and EDX imaging were prepared by precipitating and cleaning the nanodrugs in ethanol/water mixture via centrifugation (15,000 rpm, 30 min, Sorval^TM^ Legend^TM^ Micro 17 microcentrifuge) and leaving it to dry overnight under the chemical hood. The well-dried powder samples of nanodrugs were then mounted on SEM stubs using a carbon tape for imaging. A 10 kV electron beam was used for SEM imaging and EDX mapping of the nanodrug samples. 

The crystal phase of the three new nanodrug samples were investigated on a Rigaku SmartLab X-ray diffractometer (XRD) operated using a Cu source (Kα, λ = 1.54 Å). Dried powder samples of nanodrugs for XRD analysis were prepared via centrifugation and overnight drying similar to the process used for SEM samples. XRD measurements were conducted in the 2θ range of 10–90°. 

A Nicolet iS50 (Thermo Fisher Scientific) fourier transform infrared spectroscopy (FTIR) equipped with mid and far-IR capable diamond attenuated total reflectance (ATR) stage was used to investigate the surface functional groups of the nanodrugs. Powder samples of the three nanodrugs including iron oxide, zinc oxide, and combined were prepared using a process similar to that used for the XRD and SEM samples. All FTIR measurements were conducted at room temperature in the wavenumber range of 650–4000 cm^−1^. Results were reported as an average of three consecutive measurements. The multimodal material characterization helps in understanding the effects of size, surface charge, and morphology of the nanodrugs on their transport behavior. 

### 2.4. Description of the Two Flow Channels

The flow path encountered by the nanoscale drug molecules during in vivo transport contains various biomolecules that induce surface roughness in the flow path. For example, proteins and phospholipids in pulmonary systems and blood vessels or protein molecules in vascular systems can contribute towards the overall surface roughness of the flow path. Two different flow channels were prepared to mimic the rough flow path and in vivo microenvironments for realizing a reliable in vitro experimental approach to analyze the transport of different nanodrugs. The flow channels were constructed using thin polyethylene tubes with an outer diameter of 5 mm, thickness of 1 mm, and a length of 15.20 cm. Two rectangular objects of varying dimensions were prepared via 3D printing for addition within the polyethylene tubes to induce surface roughness. These rectangular objects of specific dimensions were designed using SolidWorks 3D printing software (2023 ver). 3D printing was performed on a Makerbot Replicator 2 Desktop 3D printer using PLA polymer filament. A 3D printed rectangular structure (bump) of dimensions 4 mm × 4 mm × 7 mm was inserted within the polystyrene tube at a distance of 2.5 cm from the inlet for the first flow channel. The rectangular object was placed within the thin cylindrical tube at a distance of 7.6 cm from the inlet for forming the second flow channel. The two flow channels were subsequently used for flow experiments to understand the transport of nanodrugs. 

### 2.5. Transport Experiments

The three different metal oxide nanodrugs synthesized including iron oxide, zinc oxide, and combined Cu-Zn-Fe oxide were used as model nanodrugs for the transport experiments. Each of the nanodrugs was investigated using the two model biomimetic flow channels that were prepared for this study. The transport experiments were also conducted with seven different target concentrations (e.g., 0.011 gL^−1^, 0.056 gL^−1^, 0.284 gL^−1^, 0.583 gL^−1^, 1.581 gL^−1^, 3.690 gL^−1^, and 11.070 gL^−1^) of each nanodrug to investigate the forces dominating the flow of nanodrugs in this regime. All measurements of transport properties like flow velocity and mass loss of nanodrugs during transport through the flow channels were reported as an average of three consecutive runs, keeping all experimental conditions the same. In a typical transport study of the nanodrugs, 1 mL aliquot of the aqueous nanodrugs at the target concentration was manually injected through the flow channel using a syringe mimicking manual administration of drugs to the patient. The time required for the nanodrug to flow through the entire length of the channel was monitored to determine the average flow velocity of the nanodrugs. The nanodrug was also collected in a vial at the end of the flow path and weighed to determine the mass loss of nanodrugs during the transport process. Error bars are reported based on standard deviation for both the velocity and mass loss data. This experimental transport study using the flow channels mimicking the rough surfaces of vascular or pulmonary network is used as a facile experimental platform to predict the transport of new nanodrugs for applications in drug delivery. 

## 3. Results and Discussion

**Figure 1:**
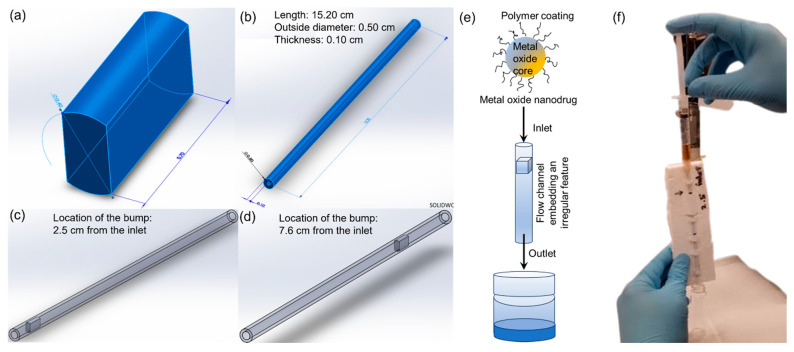
Flow experiment to understand the transport of nanodrugs through rough surfaces: (**a**) schematic of the bump, (**b**) schematic of the flow tube, (**c**) schematic of the flow channel 1 developed for the nanodrug flow experiments, (**d**) schematic of flow channel 2, (**e**) schematic of the flow experiment, and (**f**) photo of the flow experiment.

A few typical delivery paths for nanodrugs are human vasculature and pulmonary arteries. These structures comprise of anatomical irregularities from epithelial lining and contain several biomolecules, platelets, and blood cells that make the surfaces physiologically rough. Therefore, new flow channel assemblies designed to mimic rough physiological surfaces were used to investigate the flow of nanodrugs in this study. A 3D printed rectangular structure or bump served as the irregular feature for realizing the two new flow channels mimicking a vasculature (Figure 1a). The flow channels consisted of the rectangular bump inserted within a hollow plastic tube of length 15.2 cm, diameter 0.5 cm, and thickness 0.1 cm (Figure 1b). Two different flow channel configurations were developed by placing the rectangular structure at 2.5 cm and 7.6 cm from the inlet of the tube, respectively (Figure 1c,d). Figure 1e,f show the schematic and an image of a typical flow experiment where aqueous suspension of the nanodrug was injected through the flow channel assembly to mimic drug delivery via manual injection while the flow velocity and mass loss of the nanodrug during the transport were closely monitored. Three different nanodrugs of varying size and composition were used for the flow studies while each type of the nanodrug was tested at seven different concentrations. 

**Figure 2:**
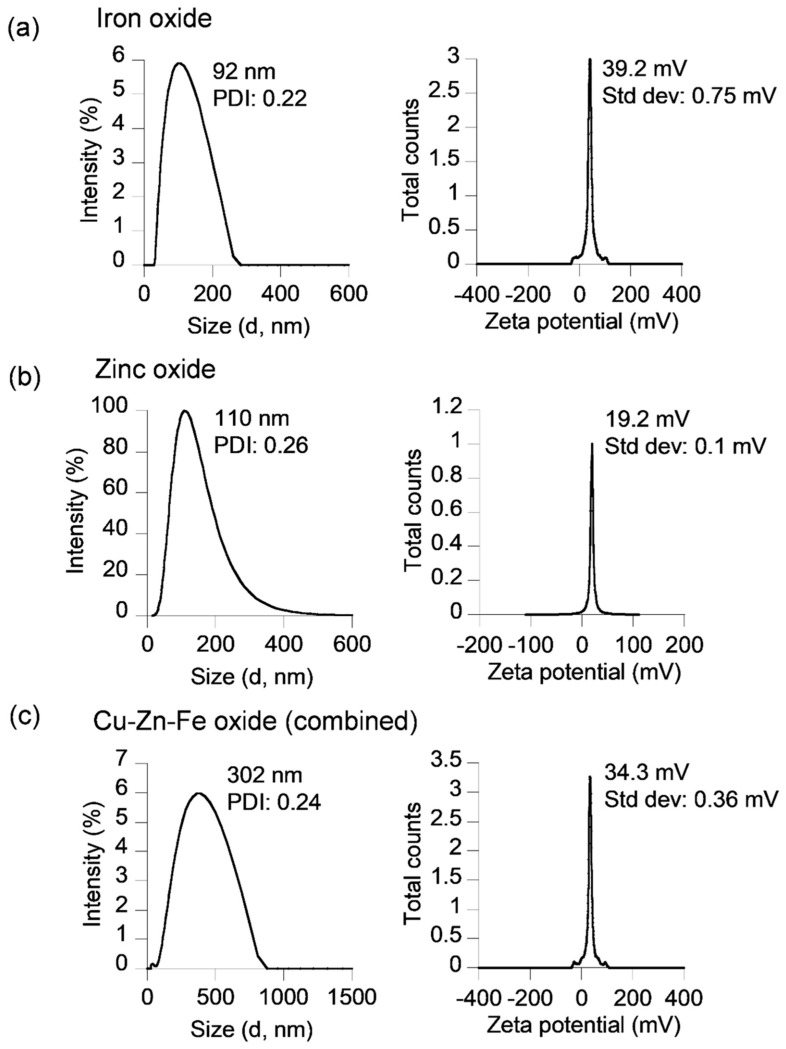
DLS size and zeta potential plots of the different nanodrugs used in the flow experiments: (**a**) iron oxide nanodrugs, (**b**) ZnO nanodrugs, and (**c**) Cu-Zn-Fe oxide nanodrugs or combined nanodrugs.

All three compositions of the nanodrug were synthesized via a modified polyol method to directly render them water-soluble in one step. Iron oxide nanodrugs, coated with a PVP/PEI ligand mixture, served as the first nanodrug for the flow studies. The aqueous-phase iron oxide nanodrugs had a hydrodynamic size of 92 ± 0.22 nm, based on the intensity-weighted DLS measurements, while the zeta potential measurements showed a stable and positively charged surface (ζ = 39.2 ± 0.75 mV) for this nanodrug (Figure 2a). The hydrophilic ligand (PVP/PEI)-coated zinc oxide nanodrug composition showed a slightly higher hydrodynamic size of 110 ± 0.26 nm and a positive surface charge of 19.2 ± 0.1 mV (Figure 2b). However, the lower absolute value of zeta potential of this nanodrug indicated that the surface was stabilized via steric hindrances from the ligands instead of electrostatic stabilization. The third nanodrug composition consisted of a combination oxide of Cu, Zn, and Fe as the nanodrug core with an outer ligand shell of PEI. The aqueous phase combined nanodrug sample was larger than the other two nanodrugs with a hydrodynamic size of 302 ± 0.24 nm and a stable positively charged surface (ζ = 34.3 ± 0.36 mV) owing to the hydrophilic polymer coating (Figure 2c). 

**Figure 3:**
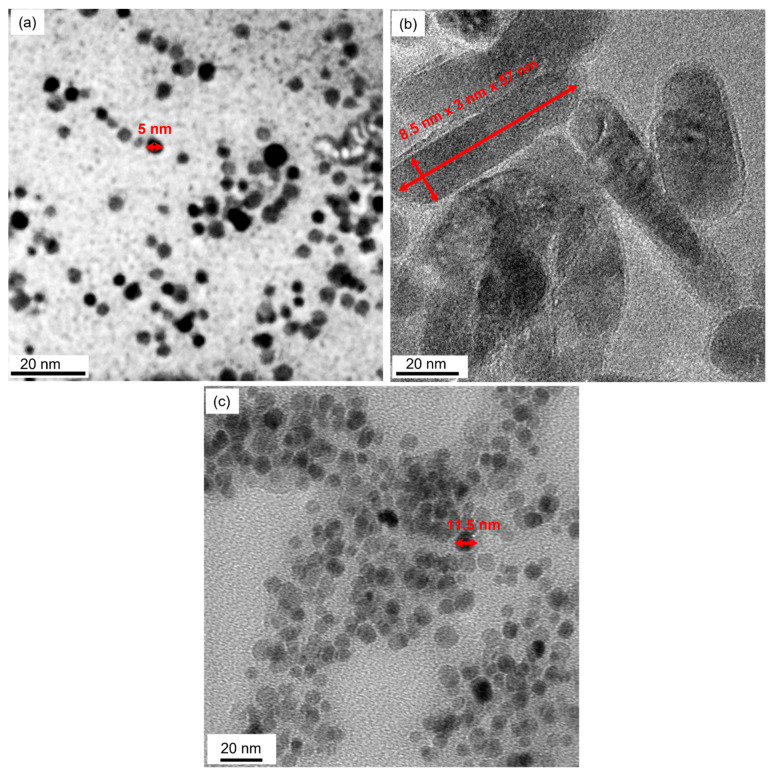
TEM images of the nanodrugs: (**a**) Iron oxide (**b**) zinc oxide, and (**c**) combined.

These nanodrugs were primarily spherical in shape as seen from the representative TEM image of the iron oxide nanodrugs (Figure 3a). The core of the spherical iron oxide nanodrugs were ~5 nm in size from the TEM image. Zinc oxide nanodrugs, on the other hand, were shaped like thin rods with a core size of ~9 nm × 3 nm × 57 nm, which was higher than that of the iron oxide nanodrugs (Figure 3b). The core of the combined nanodrugs consisting of Cu-Zn-Fe oxide was ~12 nm in size and was larger than the iron oxide nanodrugs, which confirms with the previous observations from our DLS measurements (Figure 3c). The combined nanodrugs were also spherical in morphology. Sizes of the respective nanodrugs are shown with marked arrows on the TEM images. Recently, Mn-based mixed metal ferrite nanostructures have also shown sphere-like octahedral morphology [42]. 

**Figure 4:**
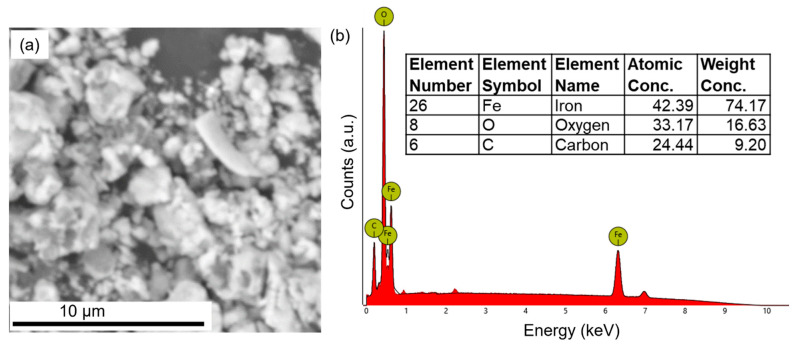
SEM and EDX characterization of iron oxide nanodrugs: (**a**) SEM image and (**b**) EDX plot.

The chemical compositions of the three nanodrugs were analyzed via SEM-EDX. Figure 4 shows the SEM and EDX characterization of iron oxide nanodrugs. The EDX spectrum clearly shows the presence of Fe, O, and C in this nanodrug sample (Figure 4b). The C peak in the EDX plot comes from the carbon tape used to mount the dried powder sample of the nanodrug on the SEM stub while the Fe and O is attributed to the composition of the nanodrug. Therefore, the EDX spectrum supported the expected iron oxide chemical composition of this nanodrug. The average elemental composition, based on five different EDX spectra from various regions of the sample, was determined to be Fe_1.27_O, which was close to the stoichiometric amounts used during the synthesis (Figure 4b).

**Figure 5:**
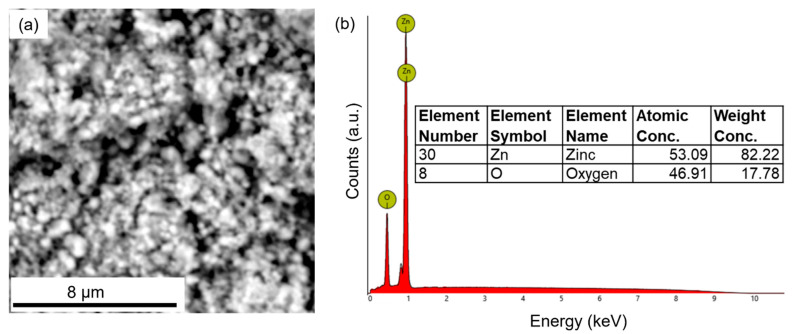
SEM and EDX characterization of zinc oxide nanodrugs: (**a**) SEM image and (**b**) EDX plot.

Figure 5a shows the SEM image of zinc oxide nanodrugs. The EDX spectrum of zinc oxide nanodrugs supported the presence of Zn and O in the sample, as expected (Figure 5b). The elemental composition of the nanodrugs was determined to be Zn_1.13_O, on the basis of SEM-EDX spectra from five different regions of the sample. The composition shows a close match to the stoichiometric quantities used for the preparation of this nanodrug. 

**Figure 6:**
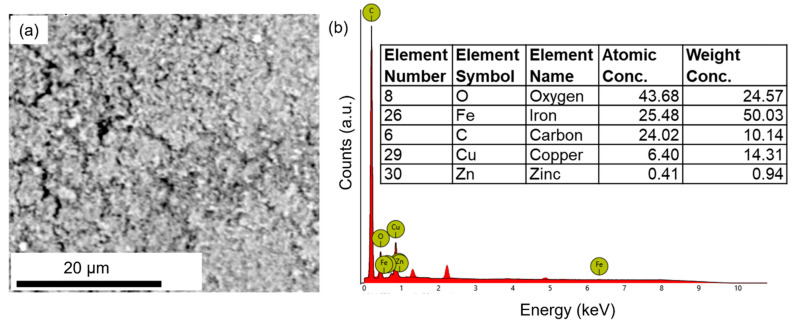
SEM and EDX characterization of combined Cu-Zn-Fe oxide nanodrugs: (**a**) SEM image and (**b**) EDX plot.

The SEM image and EDX spectrum of the combined Cu-Zn-Fe oxide nanodrugs also showed the presence of Cu, Zn, Fe, and O in the sample, as expected (Figure 6a,b). The carbon peak in the EDX spectrum is attributed to the carbon tape on the SEM stub used for loading the sample. However, the elemental composition showed a difference from the stoichiometric amounts used during the synthesis, i.e., CuZnFe_4_O_6_ (Figure 6b). 

**Figure 7:**
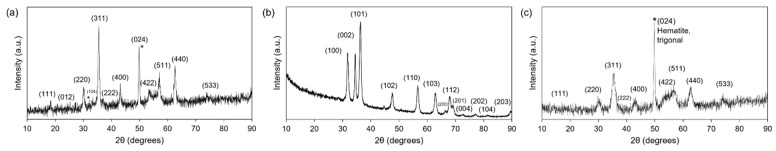
XRD characterization of nanodrugs: (**a**) iron oxide, (**b**) zinc oxide, and (**c**) combined Cu-Zn-Fe oxide.

Figure 7a shows the XRD profile for the iron oxide nanodrugs. The crystal phase of the nanodrug primarily consisted of cubic magnetite (space group: $\mathrm{Fd}\bar{3}m$, 227) with presence of the trigonal hematite phase (space group: $R\bar{3}c$, 167), based on the ICSD database. The diffraction peaks at 2θ values of 18.5°, 25.0°, 30.1°, 35.4°, 37.1°, 43.1°, 53.4°, 57.0°, 62.5°, and 74.0° closely matched with the (111), (012), (220), (311), (222), (400), (422), (511), (440), and (533) crystal planes of magnetite while the 2θ peaks at 33.3° and 49.6° corresponded to the (104) and (024) crystal planes of the hematite phase. A pure wurtzite phase (hexagonal, space group: P6_3_mc (186)), was observed for the zinc oxide nanodrugs, as suggested by the JCPDS database (card no. 36-1451) [43]. The diffraction peaks in the XRD profile of zinc oxide nanodrugs at 2θ values of 31.8°, 34.5°, 36.3°, 47.6°, 56.7°, 63.0°, 67.0°, 68.1°, 69.2°, 72.8°, 77.1°, 81.8°, and 89.6° corresponded to the (100), (002), (101), (102), (110), (103), (200), (112), (201), (004), (202), (104), and (203) crystal planes of the wurtzite phase (Figure 7b). The crystal structure of combined Cu-Zn-Fe oxide nanodrugs was primarily composed of cubic Cu_4_Zn_6_Fe_2_O_4_ phase (space group: $\mathrm{Fd}\bar{3}m$, no. 227; pdf card # 01-077-0013) with some presence of a trigonal hematite phase (space group: $R\bar{3}c$, no. 167) (Figure 7c). The XRD peaks corresponding to 2θ angles of 18.2°, 30.1°, 35.7°, 37.0°, 43.0°, 53.4°, 56.9°, 62.5°, and 73.9° could be indexed to the (111), (220), (311), (222), (400), (422), (511), (440), and (533) crystal planes of the cubic Cu_4_Zn_6_Fe_2_O_4_ phase, based on the ICSD database, while the peak at 49.9° corresponding to the (024) plane of hematite showed the presence of the hematite phase. Rietveld refinement for the XRD measurements of the respective nanodrugs is shown in Figures S2–S4 (Supplementary Materials). 

**Figure 8:**
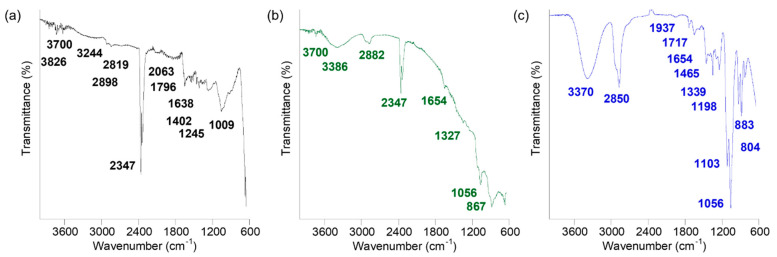
FTIR plots: (**a**) iron oxide nanodrug, (**b**) zinc oxide nanodrug, and (**c**) combined nanodrug.

Surface functionalization plays a major role on the overall size and surface charge of the nanodrugs, which in turn influences their transport properties. Therefore, the surface chemical groups of the iron oxide, zinc oxide, and combined nanodrugs were investigated via FTIR spectroscopy over the spectral range of 650–4000 cm^−1^ and presented in Figure 8. For the iron oxide nanodrugs, the FTIR peaks at 3826 cm^−1^ and 3700 cm^−1^ could be attributed to the double absorption from NH_2_ bonds in PEI while the peak at 3244 cm^−1^ was attributed to hydroxyl vibrations of PVP (Figure 8a) [44,45]. The peak at 2898 cm^−1^ was attributed to the asymmetric CH_2_ groups of the pyrrole ring in PVP while the 2819 cm^−1^ peak corresponded to the stretching and vibrations of the CH_2_ groups in PEI. The peak around 2300 cm^−1^ was due to CO_2_ from the atmosphere. The presence of a peak at 2063 cm^−1^ in the iron oxide nanodrug sample could indicate the presence of a C=C=N ketenimine bond from the attachment of PVP and PEI on the surface of the nanodrug. The peaks at 1790 cm^−1^ indicated the presence of C=O while the peak at 1638 cm^−1^ was due to N-H bending from PEI. The peaks at 1402 cm^−1^, 1245 cm^−1^, and 1009 cm^−1^ corresponded to the C-H stretch, C-N bonds, CH_2_ groups of PVP ligand on the surface of the nanodrug. Therefore, the presence of the ligand mixture of PVP and PEI on the surface of iron oxide nanodrugs was clearly visible from the FTIR profile. Similarly, the FTIR spectra of zinc oxide nanodrugs also indicated the presence of both ligands, PVP and PEI on the surface of the nanodrug, as expected from the synthesis (Figure 8b). For example, the double absorption at 3700 cm^−1^ and 3386 cm^−1^, and the peak at 2882 cm^−1^ were due to the amino groups of PEI and the CH_2_ bonds from the pyrrole group of PVP, respectively. Atmospheric carbon dioxide during the measurements contributed to the double peaks at 2347 cm^−1^. The peak at 1654 cm^−1^ was characteristic of the N-H bending of PEI while the peaks at 1327 cm^−1^ and 1056 cm^−1^ were characteristic of the C-H and CH_2_ bonds of PVP, respectively. The peak at 867 cm^−1^ is attributed to the metal-oxygen i.e., Zn-O bonds [46,47]. Figure 8c shows the FTIR spectral features of the combined nanodrugs, which were synthesized with a PEI coating. The existence of PEI is seen from the characteristic peaks of amine at 3370 cm^−1^ along with the vibrations from CH_2_ stretching and bending of amines at 2850 cm^−1^. The presence of PEI as a ligand coating on the combined nanodrugs was also indicated by the FTIR peak at 1654 cm^−1^, corresponding to the bending of NH_2_ [48]. The peak at 1465 cm^−1^ was assigned to the symmetric bending mode of NH_3_^+^ from surface functionalization of PEI onto the combined nanodrug surfaces [48]. The peaks at 1198 cm^−1^, 1103 cm^−1^, and 1056 cm^−1^ were due to C-N stretching from the attachment of PEI on the surface of the combined nanodrugs. The FTIR peaks in the 800 cm^−1^ spectral range were attributed to the metal-oxygen bonds of the Cu-Zn-Fe oxide nanodrugs. 

**Figure 9:**
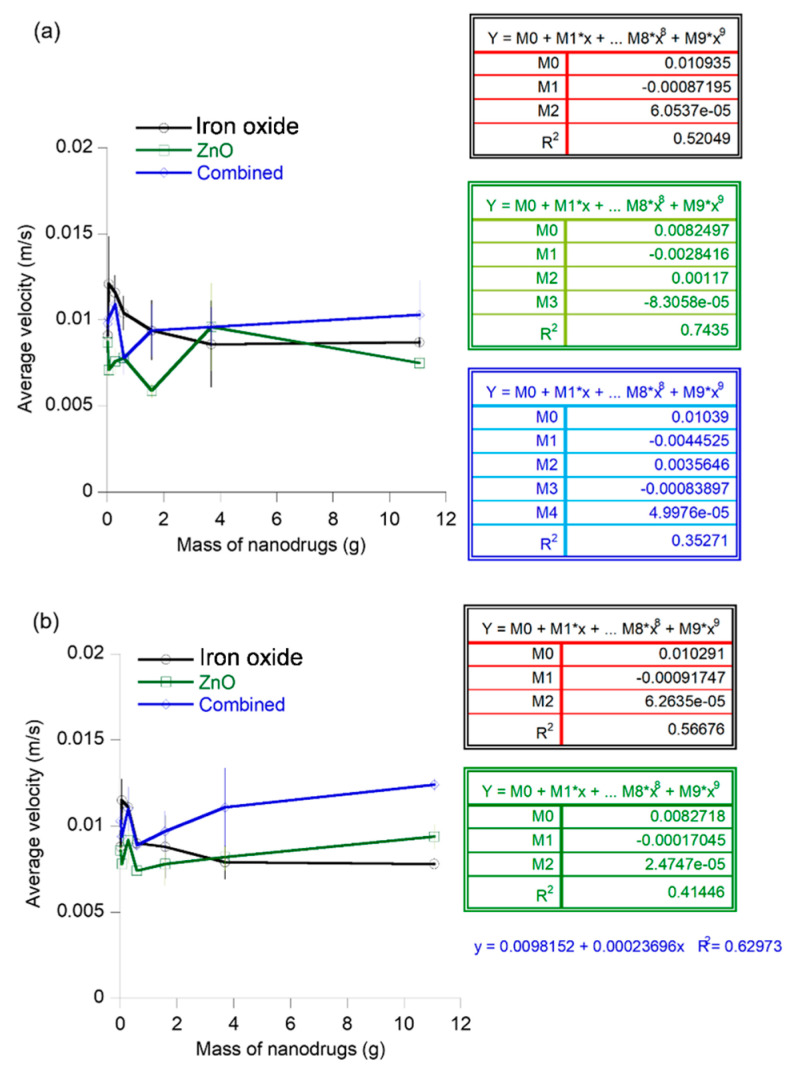
Concentration-dependent variation in the average velocity of the different nanodrugs: (**a**) velocity trend for flow channel 1 with a rectangular structure located in the flow path at 2.5 cm from the inlet and (**b**) velocity profile of the nanodrugs for flow channel 2 containing a rectangular structure at 7.6 cm from the inlet.

The three types of metal oxide nanodrugs served as the model for hydrophilic nanodrugs of spherical shapes in the new flow experiments that were developed to understand the transport characteristics of nanodrugs such as the flow velocity and deposition on the walls of the flow path. The effect of nanodrug concentration on the final velocity of the nanodrug was investigated to understand dose-dependent variations in the flow of nanosized drug molecules. The concentration-dependent effect was monitored using two different biomimetic flow channel configurations built in-house to replicate realistic vascular environments that contain various structures in the path of flow of the drug molecule. Each nanodrug composition was diluted in DI water to obtain the target mass concentrations (e.g., 0.011, 0.056, 0.284, 0.583, 1.581, 3.690, and 11.070 g). The iron oxide nanodrugs showed a decrease in average velocity with increasing concentration up to a mass concentration of 3.69 g for the first flow channel containing a rectangular bump at 2.5 cm from the entry position of the nanodrug. The velocity showed an increasing trend with concentration beyond this range (Figure 9a). Therefore, the average flow velocity of a nanodrug of this composition and size varied as a second order polynomial function (R^2^ = 0.52) with respect to dose concentrations for the first flow channel, based on our experimental results. Alternately, the slightly larger zinc oxide nanodrugs showed a concentration-dependent effect of the average flow velocity that could be best represented through a third order polynomial with a regression value of 0.74. The combined Cu-Zn-Fe oxide nanodrugs were the largest among the nanodrugs. The average velocity of this nanodrug through the first biomimetic channel showed an increasing trend with concentration beyond a mass concentration of 0.056 g, although a general velocity-concentration trend was difficult to obtain for the entire range of concentration for these nanodrugs. The second flow channel was developed by adding a rectangular structure within the flow path at a distance of 7.6 cm from the inlet. The smallest nanodrug, iron oxide, showed a gradual decrease in the average velocity with increasing concentration of the nanodrug following a second order polynomial trend (R^2^ = 0.57) for flow through the second channel (Figure 9b). In comparison, the two larger nanodrugs, ZnO and the combined nanodrugs, showed an increasing trend of the average velocity with nanodrug concentration, beyond the mass concentration of 0.284 g. The combined nanodrugs exhibited a linear velocity-concentration relation with a regression value of 0.63. 

**Figure 10:**
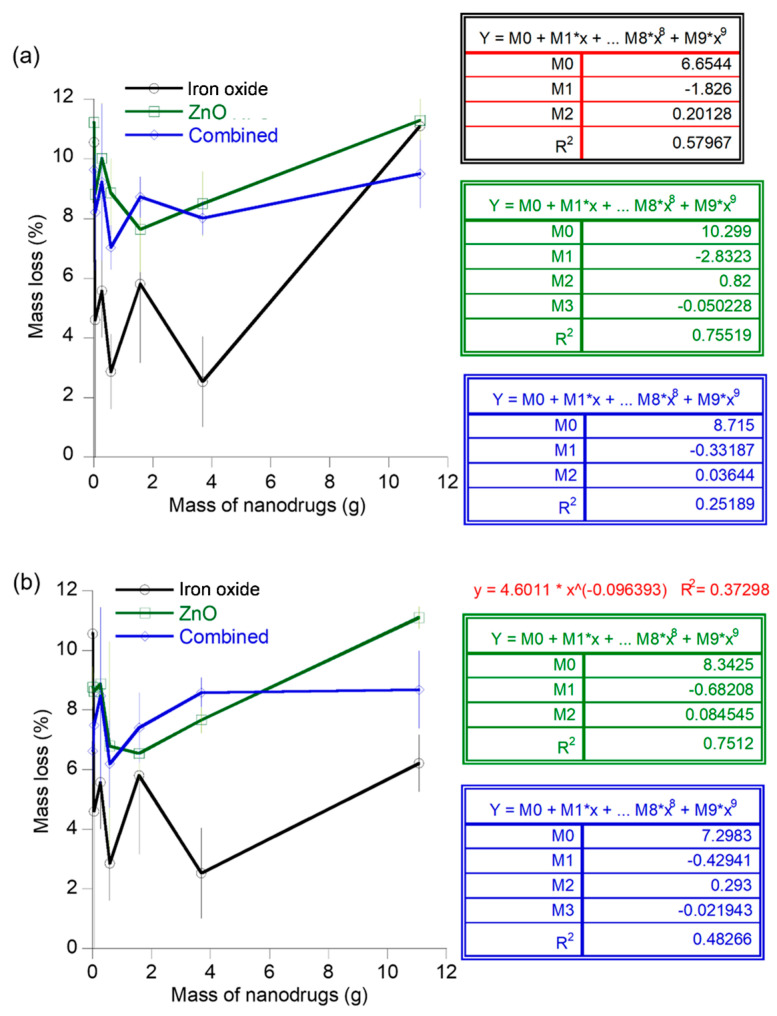
Mass loss of different nanodrugs during transport through the channels at different initial nanodrug concentrations: (**a**) mass loss of the nanodrugs for flow channel 1 and (**b**) mass loss of the nanodrugs for flow channel 2.

The mass of the nanodrugs lost during transport through the flow channels was monitored in this experiment to serve as an estimate of the quantity of doses that will be lost due to factors such as deposition on the endothelial walls and the doses that will successfully reach the target site. The mass loss of iron oxide nanodrugs showed a second order polynomial function (R^2^ = 0.58) with respect to the starting concentrations for flow through channel 1. The mass loss of the zinc oxide nanodrug compositions varied as a third order polynomial function of the nanodrug concentrations for this flow path with a regression value of 0.76. The mass loss of the combined nanodrugs showed a decreasing trend with respect to concentration beyond the concentration of 0.583 g (Figure 10a). In contrast, a decrease in the average mass loss was observed for the flow of iron oxide nanodrugs through the second type of channel up to a concentration of 0.583 g, while it was challenging to predict a general trend for the entire concentration range of the nanodrug (Figure 10b). The slightly larger zinc oxide nanodrugs exhibited a third order polynomial function (R^2^ = 0.75) of average mass loss during flow through channel 2 with respect to the starting concentrations of the nanodrug. In addition, the mass loss of ZnO nanodrugs nearly increased as a linear function of nanodrug concentration in the concentration ranges of 1.58 g to 11.07 g. The largest nanodrug out of the three types, i.e., the combined nanodrugs, also exhibited a third order polynomial trend (R^2^ = 0.48) of mass loss with respect to the initial nanodrug composition for the second flow channel. The mass loss of these nanodrugs showed a progressive increase with an increase in initial concentration of the nanodrug from 0.583–0.690 g. 

**Figure 11:**
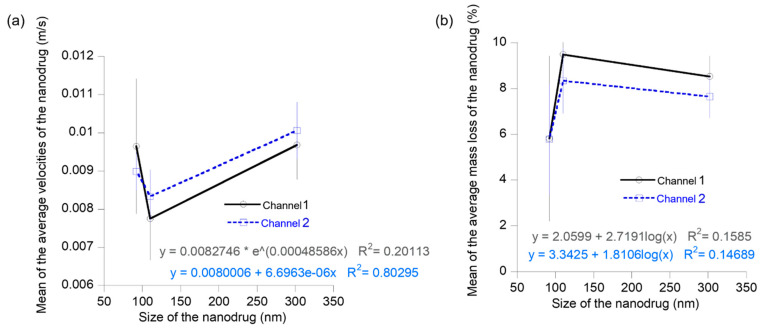
Plots showing the variation of average velocity and mass loss of the nanodrugs during transport as a function of size of the nanodrug: (**a**) average velocity plot and (**b**) average mass loss plot.

The effect of size on the transport of nanodrugs was analyzed using experimental data from the flow velocity and mass loss of the three different nanodrugs flowing through two different biomimetic flow channels (Figure 11). The mean velocity of the nanodrugs over the different concentrations showed a decrease from the 92 nm to 110 nm sizes and an increase from 110 nm to 302 nm sizes for both the flow channels (Figure 11a). The mean mass loss of the nanodrugs exhibited an opposite trend as compared to the velocity. It increased from 92 nm to 110 nm sizes of the nanodrugs and decreased from 110 nm to 302 nm for both types of flow channel (Figure 11b). Transport of these nanodrugs through the biomimetic flow channels containing customized surface features in the flow path can be affected by diffusion, convection, and deposition of particles via sedimentation. The trends in velocity and mass loss provide key insights on the dominant mechanisms controlling the flow of nanodrugs under these flow conditions. The decreasing velocity as the size of the nanodrug increases from 92 nm to 110 nm suggests that the flow of nanodrug could be diffusion-controlled in this size range. Diffusivity of spherical particles in a fluid is an inverse function of particle diameter, based on the Stokes–Einstein equation. Particles with high diffusivity exhibit a low deposition rate [33]. The mass loss of nanodrugs during the flow through channels 1 and 2 can be attributed to the deposition of particles on the walls of the flow channels and therefore, provide a measure of the deposition rate of the nanodrugs. The corresponding increase in the mass loss or deposition rate of the nanodrugs from 92 nm to 110 nm indicate a lower diffusivity of the zinc oxide nanodrug compared to the 92 nm sized iron oxide nanodrug, confirming our analysis of a diffusion-controlled flow from the velocity results. In the cases of particle flow in fluids that are dominated by deposition induced from gravitational sedimentation, the deposition or sedimentation rate is directly proportional to d^2^, where d is the particle diameter [49]. The increasing velocity trend as the size of the nanodrug increases from 110 nm to 302 nm indicate a decreasing influence of diffusion and some influence of sedimentation-controlled deposition in this size range for the flow of nanodrugs through the two custom-developed biomimetic flow channels. The mass loss, however, shows a slight decrease in this size range, which implies that competing diffusion and sedimentation mechanisms control the nanodrug transport in this size regime. The decrease in deposition for the 302 nm sized nanodrugs can be explained in terms of an increase in diffusivity of the combined nanodrug as compared to the zinc oxide nanodrug, as seen from the trend in the velocity profile [33,39,40,49].

**Figure 12:**
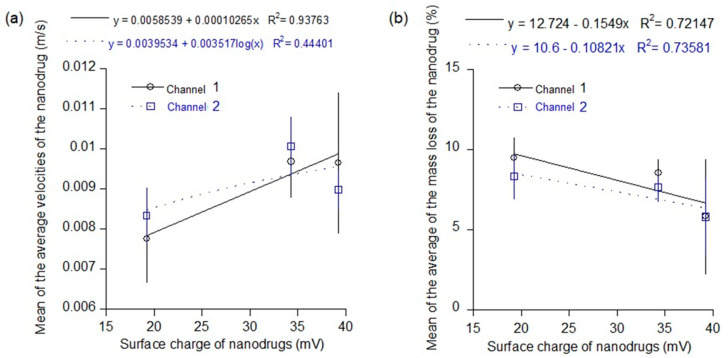
Plots showing the variation of average velocity and mass loss of the nanodrugs during transport as a function of surface charge of the nanodrug: (**a**) average velocity plot and (**b**) average mass loss plot.

Surface properties such as the surface charge of the nanodrugs also have a major influence on their transport. For example, positively charged nanoparticles fused readily within the oppositely charged cell membrane while the negatively charged nanoparticles were not as readily transported within the cell membrane due to repulsion from like charges [25]. The three new nanodrugs investigated in this study were all positively charged due to the PEI ligand coating. The PEI ligand is commonly used for various drug delivery applications including gene delivery and cell adhesion. However, there were variations in the surface charges of the three nanodrugs. Therefore, Figure 12 shows the overall effect of surface charge of the nanodrugs on their average velocity and mass loss during transport through the two uneven channels. The average velocity of the nanodrugs increased linearly (regression value = 0.94) with an increase in surface charge of the positively charged nanodrugs for the first flow channel (Figure 12a). Although, variation of the flow channel had an impact on this average velocity trend of the nanodrugs, the nanodrugs with a higher positive charge generally moved faster through the flow channels. There was a consequent decrease in the mass loss during transport through both the uneven flow channels for the nanodrugs with higher surface charges (Figure 12b). The nanodrugs with higher positive charges flowed faster through the uneven channels and therefore also showed less deposition on the channel walls during the transport. This is a useful finding for applications in drug delivery and future in vivo testing of these nanodrugs.

## 4. Conclusions

In conclusion, an experimental approach was successfully developed to predict the dominating mechanisms governing transport of nanodrugs in various size ranges. New and customized flow channels containing custom built structures within the path of flow was realized to mimic a realistic environment for investigating transport of nanodrugs through paths such as the vascular network or arteries in lungs. Three different nanodrugs containing metal oxide or mixed metal oxide core and PVP/PEI-based biocompatible ligand coatings were successfully synthesized for the study via a modified polyol route. The iron oxide and combined nanodrugs were spherical in shape while a thin rod-like morphology was observed for the zinc oxide nanodrugs. FTIR measurements confirmed the respective ligand coatings of the nanodrugs. The chemical composition of the iron oxide and zinc oxide nanodrugs were close to stoichiometric ratios while the combined nanodrugs showed slight deviation from the stoichiometric composition, based on the SEM-EDX measurements. A cubic magnetite phase was exhibited by the iron oxide nanodrugs with some presence of the hematite phase. The zinc oxide nanodrugs showed a pure wurtzite phase while the combined nanodrugs exhibited a primarily cubic Cu_4_Zn_6_Fe_2_O_4_ phase with presence of the hematite phase. The transport experiments conducted with iron oxide, ZnO, and Cu-Zn-Fe oxide nanodrugs showed that the flow is diffusion-controlled for nanodrugs in the size ranges of 92–110 nm. The role of sedimentation forces become more prominent over the 110–302 nm size range of the nanodrugs. A higher surface charge of the nanodrugs induces faster transport and lower deposition on the walls of the flow channels. The results from this study will be valuable in realizing an experimental approach to predict the quantity of nanodrugs reaching a target diseased site and therefore, the dosage required for the nanodrug. This new experimental approach can serve as a support for in vivo studies to bridge the existing gap between clinical trials and clinical translation of the new nanodrugs. 

## Data Availability

Please contact the corresponding author for any data request.

## Supplementary Materials

The following supporting information can be downloaded at: https://www.mdpi.com/article/10.3390/ma16155485/s1, Figure S1: UV-vis absorbance plot of the three nanodrugs synthesized for the transport studies. Figure S2: Rietveld fit for cubic magnetite phase for the XRD data of iron oxide nanodrug. Figure S3: Rietveld fit for wurtzite phase for the XRD data of zinc oxide nanodrug. Figure S4: Rietveld fit for cubic Cu_4_Zn_6_Fe_2_O_4_ phase for the XRD data of Cu-Zn-Fe oxide (combined) nanodrug.
