# Supplementary material for: A Novel Experimental Approach to Understand the Transport of Nanodrugs
Source: Materials (Basel). 2023 Aug 5;16(15):5485. doi: 10.3390/ma16155485 (PMC10419439; doi:10.3390/ma16155485)
Supplement: Supplementary file 1 [file materials-16-05485-s001.zip › materials-2484964-supplementary.pdf]

Supporting Information

Article

A Novel Experimental Approach to Understand the Transport of Nanodrugs

Soubantika Palchoudhury [1,*], Parnab Das [2], Amirehsan Ghasemi [3], Syed Mohammed Tareq [3], Sohini Sengupta [1], Jinchen Han [1], Sarah Maglosky [1], Fajer Almanea [1], Madison Jones [1], Collin Cox [1] and Venkateswar Rao [1]

[1] Chemical and Materials Engineering, University of Dayton, Dayton, OH 45469, USA
[2] Civil, Construction and Environmental Engineering, The University of Alabama, Tuscaloosa, AL 35487, USA
[3] Civil and Chemical Engineering, University of Tennessee, Knoxville, TN 37996, USA

* Correspondence: spalchoudhury1@udayton.edu

Figure S1 shows the ultraviolet-visible spectroscopy (UV-vis) characterization of the iron oxide nanodrugs, zinc oxide nanodrugs, and combined Cu-Zn-Fe oxide nanodrugs synthesized via a modified polyol method and used as model nanodrugs for the transport experiments. Aqueous aliquots of the three nanodrugs were measured on an Agilent Cary 60 UV-vis. Measurements were recorded as an average of three consecutive runs.

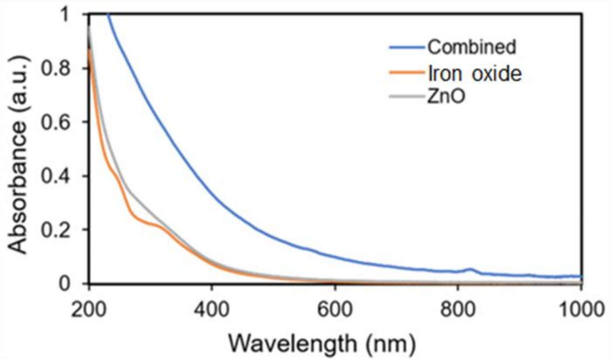

Figure S1. UV-vis absorbance plot of the three nanodrugs synthesized for the transport studies.

Figures S2-S4 show the Rietveld refinement of the XRD data of the three nanodrugs. Rietveld fit was conducted using the GSA-II software.

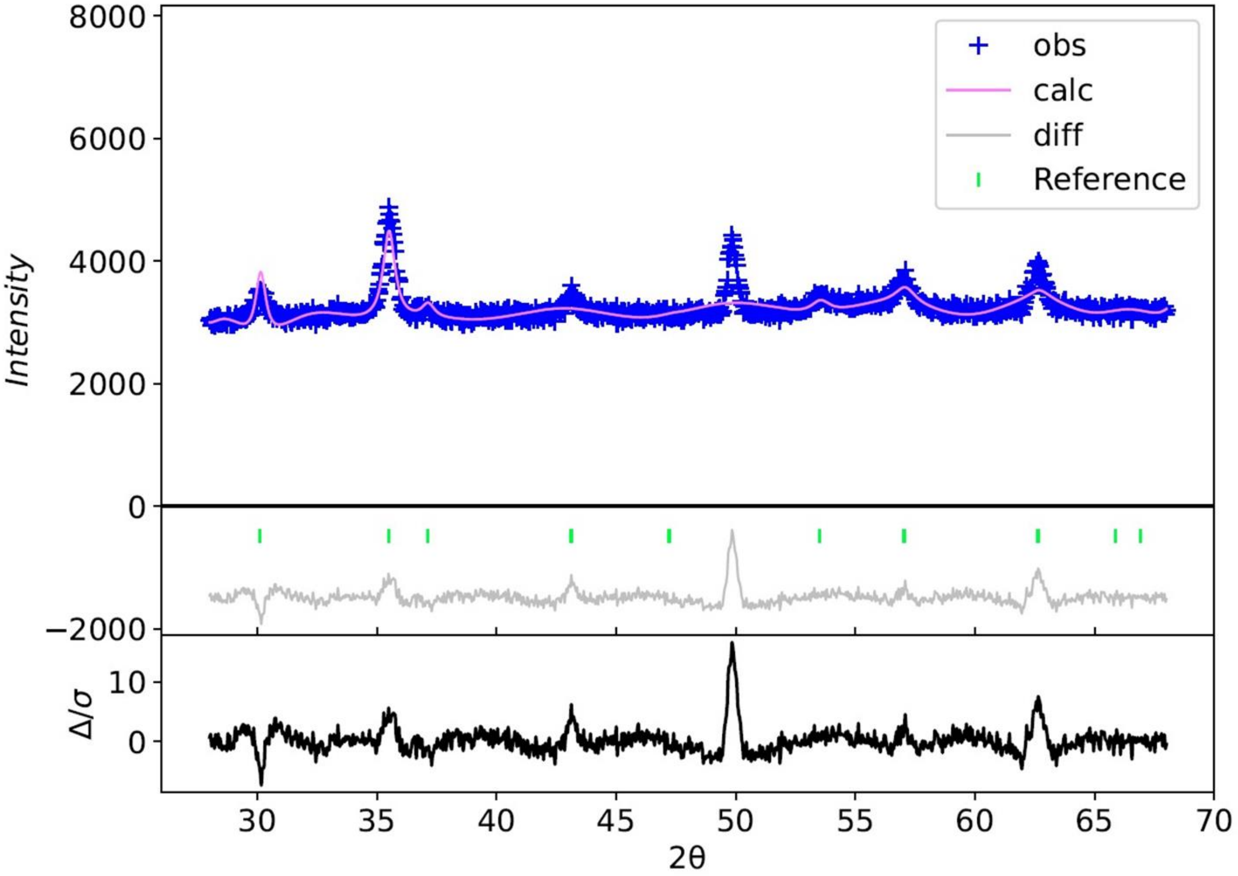

Figure S2. Rietveld fit for cubic magnetite phase for the XRD data of iron oxide nanodrug.

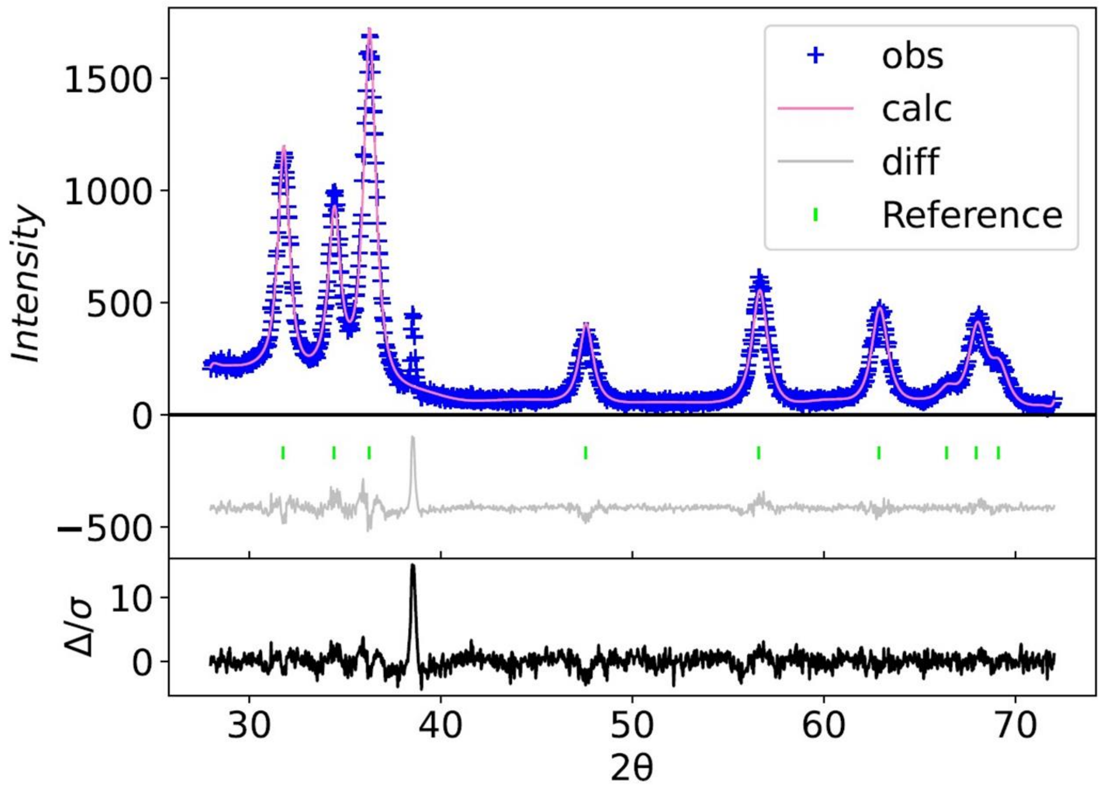

Figure S3. Rietveld fit for wurtzite phase for the XRD data of zinc oxide nanodrug.

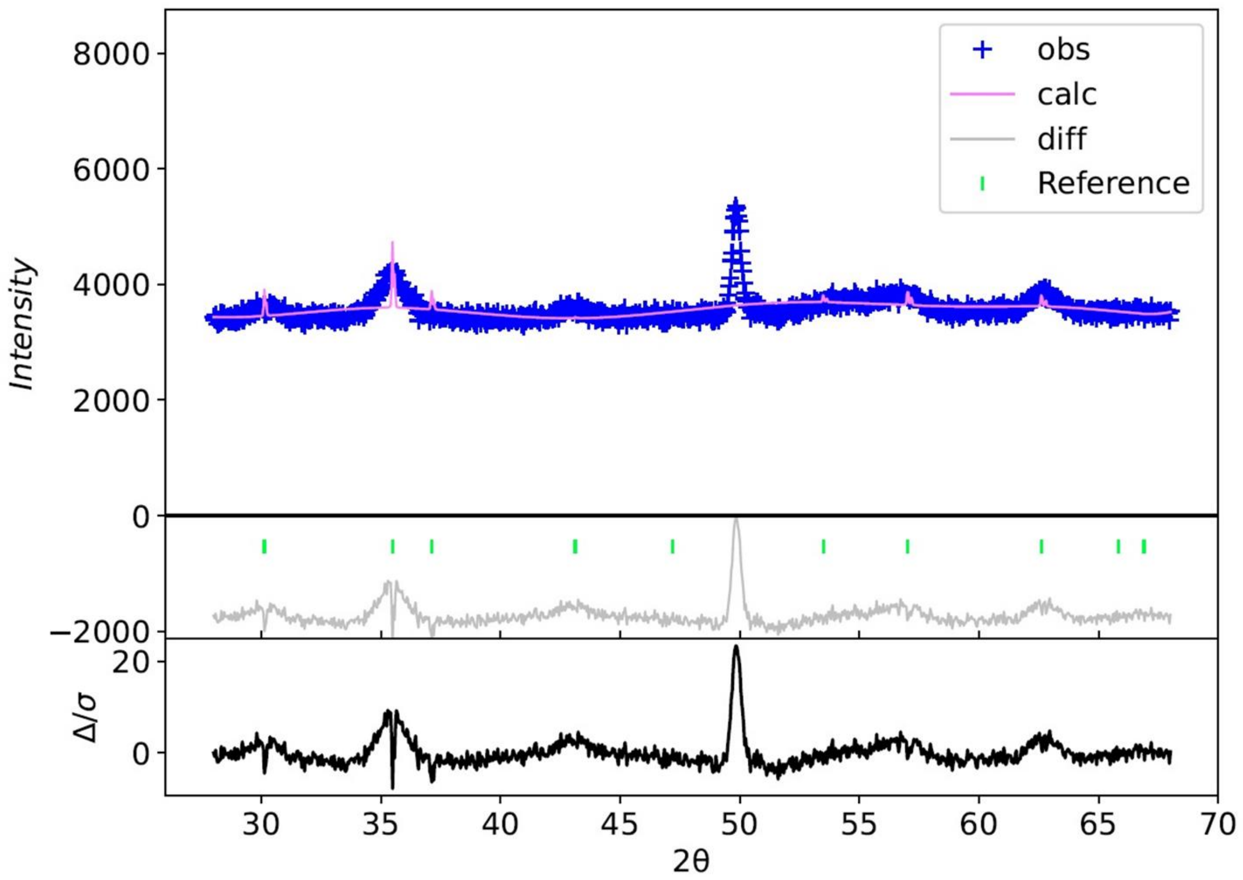

Figure S4. Rietveld fit for cubic $Cu_4Zn_6Fe_2O_4$ phase for the XRD data of Cu-Zn-Fe oxide (combined) nanodrug.
